# Combined effects of lifestyle risk factors on fatty liver index

**DOI:** 10.1186/s12876-020-01270-7

**Published:** 2020-04-15

**Authors:** Ulla Nivukoski, Markus Niemelä, Aini Bloigu, Risto Bloigu, Mauri Aalto, Tiina Laatikainen, Onni Niemelä

**Affiliations:** 1 Department of Laboratory Medicine and Medical Research Unit, Seinäjoki Central Hospital and Tampere University, Hanneksenrinne 7, 60220 Seinäjoki, Finland; 2 Faculty of Medicine, University of Oulu, 90014 Oulu, Finland; 3 Center for Life Course Health Research, University of Oulu, 90014 Oulu, Finland; 4 Infrastructure for Population Studies, Faculty of Medicine, University of Oulu, 90014 Oulu, Finland; 5 Department of Psychiatry, Seinäjoki Central Hospital and Tampere University, 33014 Tampere, Finland; 6 National Institute for Health and Welfare (THL), 00271 Helsinki, Finland; 7 The Institute of Public Health and Clinical Nutrition, University of Eastern Finland, 70210 Kuopio, Finland; 8 Joint Municipal Authority for North Karelia Social and Health Services, 80100 Joensuu, Finland

**Keywords:** Alcohol, NAFLD, Obesity, Physical activity, Steatosis

## Abstract

**Background:**

Factors of lifestyle may have a major impact on liver-related morbidity and mortality.

We examined independent and joint effects of lifestyle risk factors on fatty liver index (FLI), a biomarker of hepatic steatosis, in a population-based cross-sectional national health survey.

**Methods:**

The study included 12,368 participants (5784 men, 6584 women) aged 25–74 years. Quantitative estimates of alcohol use, smoking, adiposity and physical activity were used to establish a total score of risk factors, with higher scores indicating an unhealthier lifestyle. FLI was calculated based on an algorithm including body mass index, waist circumference, serum gamma-glutamyltransferase and triglycerides.

**Results:**

The occurrence of FLI ≥ 60% indicating fatty liver increased from 2.4% in men with zero risk factors to 81.9% in those with a total risk score of 7–8 *(p* <  0.0005 for linear trend) and in women from 0 to 73.5% *(p* <  0.0005). The most striking individual impacts on the likelihood for FLI above 60% were observed for physical inactivity *(p* <  0.0005 for both genders) and alcohol consumption (*p* <  0.0005 for men). Interestingly, coffee consumption was also found to increase with increasing risk factor scores *(p* <  0.0005 for linear trend in both genders).

**Conclusions:**

The data indicates that unfavorable combinations of lifestyle risk factors lead to a high likelihood of hepatic steatosis. Use of FLI as a diagnostic tool may benefit the assessment of interventions aimed at maintaining a healthy lifestyle and prevention of liver-related morbidity.

## Background

Excessive alcohol use, smoking, and lack of physical activity are typical risk factors of lifestyle, which may contribute to adiposity, fatty deposition in the liver and increased all-cause mortality [1–4]. Furthermore, several risk factors are often present concomitantly in the same individual [5, 6]. Recent studies have concluded that simultaneous adherence to multiple healthy lifestyle factors could significantly prolong life expectancy suggesting substantial therapeutic implications for interventions focusing on basic lifestyle factors [1, 7, 8].

In current societies, hepatic steatosis is a highly common manifestation of health problems driven by behavioral factors. Building of too much fat in the liver may lead to a wide variety of clinical symptoms ranging from asymptomatic increases in biomarkers of liver function to liver cirrhosis [2, 9–11]. Recent studies have indicated that elevated alanine aminotransferase (ALT) and gamma-glutamyltransferase (GGT) activities are common in obese individuals with mild to moderate alcohol consumption suggesting cumulative hepatotoxic effects for adiposity and alcohol use [6, 9, 10, 12–14]. Smoking together with alcohol use may also have synergistic effects in increasing the odds of abnormal GGT levels [15, 16]. The increases in liver enzymes under such conditions also appear to associate with systemic inflammation, abnormal lipid status and increased risk for both hepatic and extra-hepatic complications, including cardio- and cerebrovascular diseases [14, 17–19].

Recent advances in research on liver diseases have led to the introduction of various algorithms designed for assessing individual disease risks in a non-invasive manner. Fatty liver index (FLI) is an algorithm designed for the prediction of fatty liver, which in previous external validation studies involving comparisons with ultrasonography data, has been shown to be more accurate for the identification of fatty liver than any of the conventional biomarkers of liver function [11, 20]. So far, no data have, however, been available on the impacts of unhealthy behaviors on FLI. In this work, we aimed to investigate the individual and joint effects of various lifestyle risk factors on FLI in a large Finnish population-based cohort (the National FINRISK study) encompassing detailed records on alcohol use, smoking habits, physical activity and other health-related behavior. Improved knowledge on the associations between FLI, as a proxy for fatty liver, and various risk factors of lifestyle may be assumed to provide new tools for clinical management and counseling regarding factors of lifestyle in patients with suspected hepatic steatosis.

## Methods

### Study design

Data from a cross-sectional population health survey (The National FINRISK Study) carried out in six geographical areas in Finland in years 1997, 2002 and 2007 were used [13, 21, 22]. The material includes a nationally representative age- and gender stratified sample, which was drawn from the population register according to an international protocol [21]. Clinical examinations comprised physical measurements, laboratory analyses and detailed questionnaires encompassing alcohol intake, smoking, coffee consumption, physical activity, medical history, current health status and socioeconomic factors [21, 22]. Body mass index (BMI, kg/m^2^) was calculated as an index of relative body weight based on body weight and height, which were measured to the nearest 0.1 kg and 0.1 cm, respectively. Waist circumference (to the nearest 0.5 cm) was obtained from the measurements between the lowest rib and iliac crest while the study subject was at minimal respiration.

Data on alcohol use from the past 12 months was collected through questionnaires gathering information on the types of beverages, the frequency of consumption, and the amounts of each type of ethanol-containing standard drink (corresponding to 12 g of ethanol) [18]. Information on smoking was gathered with standardized questionnaires and the data was given as the number of cigarettes per day. Leisure-time physical activity including the number and total time used for physical exercises were registered using specifically designed structured questionnaires, as previously described [21, 22]. Coffee consumption as derived from the sets of standardized questions were expressed as the amounts of standard coffee servings (cups) per day.

The responses to each question on alcohol consumption, smoking, physical activity and coffee consumption were assigned to mutually exclusive and collectively exhaustive categories [21, 22]. The data was subsequently used to categorize the subjects into three ordinal levels to define scores for low risk (= 0), medium risk (= 1) and high risk (= 2) for each lifestyle factor, as previously described [1, 13]. For scoring alcohol consumption the currently recommended national limits of low-risk alcohol consumption were followed: 0 = no consumption; 1 = alcohol consumption between 1 and 14 (men) or 1–7 (women) standard drinks per week (low risk consumption); 2 = alcohol consumption exceeding 14 drinks (men) or 7 drinks (women) per week (high risk consumption). For smoking 0 = no smoking, 1 = 1–19 cigarettes per day, 2 = ≥ 20 cigarettes per day; for BMI 0 = < 25; 1 = ≥ 25 and <  30 (overweight); 2 = ≥ 30 (obesity). For physical activity, score = 0 refers to those with physical activity over 4 h per week; 1 = physical activity between 0.5 and 4 h per week and 2 = physical activity less than 30 min/week. The sum of the above scores provided the total number of risk factors, with higher scores indicating an unhealthier lifestyle.

The data was available from 12,368 participants (5784 men, 6584 women, mean age 49 ± 13 years, range 25–74 years) who completed the questionnaires and attended the medical examination. The study excluded individuals with any apparent clinical signs of liver disease, diabetes or abnormal oral glucose test, ischemic heart or brain disease, chronic inflammatory diseases, malignancy or active infection at the time of blood sampling. The investigation was performed with the understanding and written informed consent of each individual and was approved by the Coordinating Ethics Committee of the Helsinki and Uusimaa Hospital District. All surveys were conducted in accordance with the Declaration of Helsinki according to the ethical rules of the National Public Health Institute.

### Laboratory analyses

Serum ALT and GGT were analyzed by standard clinical chemical methods on an Abbott Architect analyzer following the instructions of the manufacturer (Abbott Laboratories, Abbott Park, IL, USA). Assays of high-sensitivity C-reactive protein (CRP) were carried out using a latex immunoassay (Sentinel Diagnostics, Milan, Italy) on Abbott Architect c8000 analyzer. Determinations of total cholesterol, high-density lipoprotein-associated cholesterol (HDL), low-density lipoprotein associated cholesterol (LDL) and total triglycerides were based on standard enzymatic methods. All laboratory tests were subjects to continuous external quality control programs organized by Labquality, Finland and CDC (Center for Disease Control and Prevention) quality assurance and standardization program for serum lipids. The cut-offs for the normal limits of the parameters were as follows: ALT (50 U/L men; 35 U/L women), GGT (60 U/L men; 40 U/L women), CRP (3.0 mg/L), cholesterol (5 mmol/L), HDL cholesterol (1.0 mmol/L men, 1.2 mmol/L women), LDL cholesterol (3.0 mmol/L), triglycerides (1.7 mmol/L).

### Fatty liver index

Fatty liver index is a predictor algorithm for fatty liver disease, which was computed based on BMI, waist circumference, triglycerides and GGT, as previously described by Bedogni and coworkers [20]. In this algorithm, FLI scores below 30 exclude fatty liver, scores below 30 and 60 remain inconclusive whereas scores of 60 and above indicate that fatty liver is present [20].

### Statistical methods

The study variables are reported as mean ± standard deviation (SD) or geometric means with 95% confidence intervals, as indicated. For parameters with skewed distributions a logarithmic transformation was performed. Comparisons between the variables were carried out using analysis of variance (ANOVA) with polynomial contrasts to reveal possible trends across the ordinally increasing risk score categories. The distribution of findings exceeding the cut-offs for FLI and other biomarkers in various risk categories were analyzed by chi-square test for trend. Multinomial logistic regression was used to estimate the odds for abnormal FLI according to the individual number of lifestyle risk factor scores, adjusting for BMI, age and coffee consumption. To evaluate the individual impact of the lifestyle risk factors as predictors of abnormal FLI (≥ 60) multivariate binary logistic regression with likelihood ratio test was performed and estimates are presented as odds ratios (OR). The differences in proportions between men and women were tested using Pearson chi-square test and Fisher’s exact test as appropriate. Correlations between the study variables were calculated using Spearman’s rank correlation coefficients. The analyses were carried out with IBM SPSS Statistics 24.0 (Armonk, NY: IBM Corp.). A *p*-value < 0.05 was considered statistically significant.

## Results

Table 1 summarizes the main clinical characteristics of the subjects classified according to the score of lifestyle risk factors and gender. Higher quantities of alcohol intake, excess body weight, higher levels of cigarette smoking and physical inactivity were found to characterize the individuals with increased risk scores. In men, there was a quadratic trend between age and ordinal lifestyle risk score categories, the highest mean ages being noted in the middle section of the risk categories *(p* <  0.01) whereas in women a linear trend was observed *(p* < 0.0005). There was also a significant association between coffee consumption and increasing risk factor scores *(p* < 0.0005 for linear trend in both genders). Among the individual components of the risk factor score, a significant association was found to exist between coffee consumption and smoking status. Coffee consumption ≥4 cups/day was found in 52.3% of non-smokers, 70.9% of those smoking 1–19 cigarettes per day and in 84.4% of those smoking ≥20 cigarettes/day (*p* < 0.0005).

**Table 1:** Main characteristics of the study population, as categorized to subgroups according to the number of lifestyle risk factor scores

Men								
Risk score	0	1	2	3	4	5	6	7–8
N (%)	168 (2.9)	740 (12.8)	1392 (24.1)	1413 (24.4)	1068 (18.5)	615 (10.6)	294 (5.1)	94 (1.6)
Age, years, mean ± SD	41.8 ± 13.8	44.1 ± 13.4	45.5 ± 13.6	46.2 ± 12.9	44.1 ± 12.2	45.4 ± 11.7	44.4 ± 11.0	43.7 ± 9.7
Alcohol use, g/day	0.0 ± 0.0	4.9 ± 6.5	7.8 ± 9.0	11.5 ± 13.8	17.0 ± 19.1	23.6 ± 26.4	34.2 ± 30.5	44.7 ± 30.5
Smoking, cigarettes/day	0.0 ± 0.0	0.3 ± 1.7	1.2 ± 3.7	3.3 ± 6.7	7.2 ± 9.5	13.1 ± 11.0	18.9 ± 11.7	23.8 ± 8.5
Body mass index	23.1 ± 1.3	23.9 ± 2.0	25.3 ± 2.7	26.6 ± 3.1	27.5 ± 4.0	28.2 ± 4.3	28.6 ± 4.9	30.8 ± 3.7
Waist circumference, cm	82.5 ± 5.7	86.0 ± 6.7	89.8 ± 8.4	94.1 ± 9.1	96.3 ± 11.3	98.7 ± 11.9	100.2 ± 12.9	105.8 ± 11.1
Physical activity, exercises/week	4.1 ± 1.8	3.4 ± 1.9	2.8 ± 1.9	2.3 ± 2.0	1.7 ± 1.7	1.4 ± 1.7	1.3 ± 2.3	0.6 ± 0.9
Coffee, cups/day	3.7 ± 2.8	3.9 ± 2.9	4.1 ± 2.8	4.7 ± 3.1	5.3 ± 3.3	5.9 ± 3.8	6.0 ± 4.1	6.7 ± 4.7
**Women**								
**Risk score**	**0**	**1**	**2**	**3**	**4**	**5**	**6**	**7–8**
N (%)	338 (5.1)	1286 (19.5)	1877 (28.5)	1596 (24.2)	923 (14.0)	391 (5.9)	139 (2.1)	34 (0.5)
Age, years, mean ± SD	39.6 ± 11.9	42.3 ± 12.7	44.0 ± 12.6	45.1 ± 12.4	44.9 ± 12.5	44.3 ± 11.1	44.5 ± 10.4	47.0 ± 11.6
Alcohol consumption, g/day	0.0 ± 0.0	2.2 ± 3.4	3.9 ± 5.5	5.3 ± 7.1	7.7 ± 8.8	13.8 ± 12.3	15.6 ± 13.2	19.4 ± 12.9
Smoking, cigarettes/day	0.0 ± 0.0	0.2 ± 1.3	1.1 ± 3.4	2.3 ± 4.9	4.8 ± 6.8	7.8 ± 8.5	14.5 ± 10.3	17.4 ± 6.8
Body mass index	22.3 ± 1.6	22.8 ± 2.4	24.1 ± 3.2	26.2 ± 4.4	28.3 ± 5.1	29.1 ± 5.7	30.3 ± 5.5	32.8 ± 3.5
Waist circumference, cm	73.6 ± 5.9	75.0 ± 7.1	78.2 ± 8.7	83.0 ± 11.2	88.5 ± 13.0	90.1 ± 13.7	93.3 ± 13.1	99.9 ± 11.1
Physical activity, exercises/week	3.8 ± 1.8	3.2 ± 2.1	2.5 ± 2.0	2.3 ± 2.0	2.0 ± 2.0	1.6 ± 1.9	0.9 ± 1.3	0.8 ± 0.9
Coffee, cups/day	3.0 ± 2.3	3.2 ± 2.4	3.6 ± 2.4	3.9 ± 2.4	4.3 ± 2.7	4.5 ± 3.0	5.4 ± 3.5	4.4 ± 3.0

**Fig. 1:**
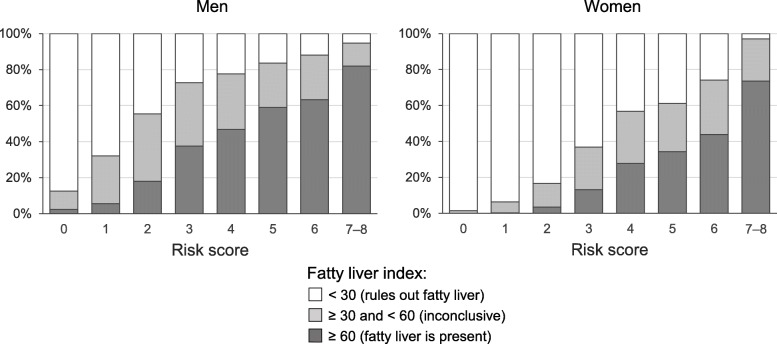
The distributions of FLI scores below 30, ≥ 30 and < 60 and 60 or above in individuals classified to lifestyle risk factor scores as follows: Alcohol consumption, 0 = no consumption; 1 = alcohol consumption 1–14 (men) or 1–7 (women) drinks/week; 2 = alcohol consumption in amounts exceeding 14 drinks (men) or 7 drinks (women) per week Smoking, 0 = no smoking, 1 = 1–19 cigarettes/day, 2 = ≥ 20 cigarettes/day. BMI, 0 = BMI < 25; 1 = BMI ≥ 25 and < 30; 2 = ≥ 30. Physical activity, 0 = those with physical activity over 4 h/week; 1 = those with physical activity 0.5–4 h/week; 2 = those with physical activity less than 30 min/week. The sum of the scores yielded a total risk factor number, with higher scores indicating an unhealthier lifestyle (maximum = 8)

The data on the clinical and laboratory parameters in subgroups with different lifestyle risk factor status are summarized in Table 2. The proportions of individuals with FLI ≥ 60 (indicating that fatty liver is present) and the percentages of individuals exceeding the reference limits in the individual components of the FLI (BMI, waist circumference, serum triglycerides and GGT) as well as in biomarkers of liver function (ALT), inflammation (CRP) and lipid status (cholesterol, HDL-cholesterol, LDL-cholesterol) are also shown. Distinct dose-response relationships were observed between the number of unfavorable risk factors, FLI levels and biomarker data in all comparisons. In those with zero risk factors FLI below 30 (ruling out fatty liver) was observed in 87.5% of men and 98.5% of women (Fig. 1). While in both genders the increase in the amount of risk factors was found to lead to a sharp increase in the prevalence of FLI 60 or above suggesting fatty liver, the changes among men were found to occur in a more sensitive manner (*p* < 0.0005 for differences in proportions) (Fig. 1).

**Table 2:** Fatty liver index (FLI), its individual components and various biomarkers of liver function, inflammation and lipid status according to lifestyle risk factor scores. The percentages show the proportions of individuals exceeding the cut-offs for each parameter

Men																
Risk score	0		1		2		3		4		5		6		7–8	
FLI	18.0 ± 12.3	2.4%	25.4 ± 17.0	5.5%	37.2 ± 22.7	18.0%	49.4 ± 25.4	37.6%	55.7 ± 27.7	46.7%	62.6 ± 27.8	59.0%	67.0 ± 26.2	63.3%	79.9 ± 21.2	81.9%
BMI, kg/m^2^	23.1 ± 1.3	0.0%	23.9 ± 2.0	21.4%	25.3 ± 2.7	54.1%	26.6 ± 3.1	73.5%	27.5 ± 4.0	76.4%	28.2 ± 4.3	78.4%	28.6 ± 4.9	83.0%	30.8 ± 3.7	100.0%
Waist, cm	82.5 ± 5.7	4.2%	86.0 ± 6.7	13.0%	89.8 ± 8.4	33.5%	94.1 ± 9.1	52.7%	96.3 ± 11.3	57.0%	98.7 ± 11.9	63.7%	100.2 ± 12.9	72.4%	105.8 ± 11.1	85.1%
GGT, U/L	20.7 (19–22)	1.8%	23.3 (22–24)	5.1%	25.6 (25–26)	5.4%	31.0 (30–32)	12.1%	35.3 (34–37)	18.7%	40.6 (39–43)	24.2%	48.6 (45–53)	36.7%	56.7 (49–65)	41.5%
Trigl, mmol/L	1.02 (1.0–1.1)	12.5%	1.06 (1.0–1.1)	14.3%	1.24 (1.2–1.3)	24.4%	1.36 (1.3–1.4)	31.2%	1.46 (1.4–1.5)	35.8%	1.60 (1.5–1.7)	41.8%	1.68 (1.6–1.8)	47.8%	2.05 (1.8–2.3)	59.6%
ALT, U/L	22.1 (20–25)	0.0%	23.4 (22–24)	2.9%	25.8 (25–27)	6.7%	28.6 (28–30)	11.5%	30.6 (29–32)	16.3%	31.4 (29–33)	15.9%	34.1 (31–38)	25.0%	43.7 (38–51)	32.0%
CRP, mg/L	0.53 (0.5–0.6)	4.9%	0.62 (0.6–0.7)	7.2%	0.81 (0.8–0.9)	11.6%	0.93 (0.9–1.0)	12.1%	1.19 (1.1–1.3)	16.9%	1.38 (1.3–1.5)	21.9%	1.74 (1.5–2.0)	31.0%	2.09 (1.7–2.6)	35.5%
Chol, mmol/L	4.94 (4.8–5.1)	45.8%	5.09 (5.0–5.2)	55.8%	5.28 (5.2–5.3)	64.5%	5.50 (5.4–5.6)	72.3%	5.49 (5.4–5.6)	70.7%	5.60 (5.5–5.7)	73.3%	5.73 (5.6–5.9)	74.8%	5.96 (5.7–6.2)	84.0%
HDL, mmol/L	1.33 (1.3–1.4)	9.5%	1.36 (1.3–1.4)	8.7%	1.29 (1.3–1.3)	15.7%	1.27 (1.3–1.3)	15.6%	1.26 (1.2–1.3)	17.5%	1.26 (1.2–1.3)	18.6%	1.25 (1.2–1.3)	20.5%	1.23 (1.2–1.3)	16.0%
LDL, mmol/L	3.03 (2.9–3.2)	49.5%	3.04 (3.0–3.1)	57.0%	3.27 (3.2–3.3)	65.8%	3.39 (3.3–3.4)	71.4%	3.39 (3.3–3.5)	70.3%	3.36 (3.3–3.5)	68.7%	3.60 (3.5–3.7)	77.9%	3.52 (3.3–3.8)	70.3%
**Women**																
**Risk score**	**0**		**1**		**2**		**3**		**4**		**5**		**6**		**7–8**	
FLI	8.4 ± 6.8	0.0%	11.1 ± 10.7	0.2%	17.0 ± 16.5	3.5%	27.9 ± 24.3	13.2%	40.6 ± 28.4	27.7%	45.3 ± 30.7	34.3%	54.5 ± 29.3	43.9%	71.1 ± 23.1	73.5%
BMI, kg/m2	22.3 ± 1.6	0.0%	22.8 ± 2.4	11.7%	24.1 ± 3.2	34.8%	26.2 ± 4.4	60.0%	28.3 ± 5.1	75.9%	29.1 ± 5.7	76.0%	30.3 ± 5.5	87.8%	32.8 ± 3.5	100.0%
Waist, cm	73.6 ± 5.9	15.4%	75.0 ± 7.1	24.3%	78.2 ± 8.7	39.1%	83.0 ± 11.2	58.7%	88.5 ± 13.0	73.8%	90.1 ± 13.7	74.9%	93.3 ± 13.1	84.9%	99.9 ± 11.1	97.1%
GGT, U/L	15.8 (15–17)	5.9%	16.3 (16–17)	4.3%	17.5 (17–18)	4.7%	19.1 (19–20)	8.9%	21.9 (21–23)	10.6%	25.2 (24–27)	17.6%	28.4 (25–32)	25.9%	33.3 (27–41)	26.5%
Trigl, mmol/L	0.87 (0.8–0.9)	5.6%	0.90 (0.9–0.9)	6.4%	0.95 (0.9–1.0)	8.0%	1.04 (1.0–1.1)	13.3%	1.12 (1.1–1.2)	17.0%	1.15 (1.1–1.2)	19.5%	1.24 (1.1–1.3)	25.9%	1.50 (1.3–1.8)	32.4%
ALT, U/L	17.0 (16–18)	3.3%	17.4 (17–18)	6.6%	17.5 (17–18)	4.4%	18.1 (18–19)	6.7%	19.3 (19–20)	9.2%	20.2 (19–22)	8.3%	22.1 (19–25)	15.3%	24.1 (17–34)	9.1%
CRP, mg/L	0.64 (0.6–0.7)	9.1%	0.76 (0.7–0.8)	10.8%	0.83 (0.8–0.9)	12.4%	1.07 (1.0–1.1)	17.7%	1.42 (1.3–1.5)	27.0%	1.43 (1.3–1.6)	26.6%	2.05 (1.7–2.5)	37.2%	2.99 (2.2–4.1)	52.9%
Chol, mmol/L	5.01 (4.9–5.1)	52.7%	5.09 (5.0–5.1)	53.3%	5.21 (5.2–5.3)	59.4%	5.32 (5.3–5.4)	62.6%	5.30 (5.2–5.4)	63.8%	5.29 (5.2–5.4)	61.4%	5.39 (5.2–5.5)	66.2%	5.67 (5.3–6.1)	76.5%
HDL, mmol/L	1.66 (1.6–1.7)	5.9%	1.62 (1.6–1.6)	7.7%	1.60 (1.6–1.6)	10.4%	1.55 (1.5–1.6)	13.6%	1.50 (1.5–1.5)	19.8%	1.50 (1.5–1.5)	21.9%	1.50 (1.4–1.6)	20.3%	1.34 (1.2–1.4)	26.5%
LDL, mmol/L	2.76 (2.7–2.9)	43.0%	2.87 (2.8–2.9)	43.7%	2.96 (2.9–3.0)	50.8%	3.07 (3.0–3.1)	57.0%	3.11 (3.0–3.2)	57.7%	3.08 (3.0–3.2)	55.6%	3.15 (3.0–3.3)	60.4%	3.29 (2.9–3.7)	72.7%

The values are expressed as mean ± SD (FLI, BMI, waist circumference) or as geometric means and 95% confidence intervals (GGT, triglycerides, ALT, CRP, cholesterol, HDL, LDL)

*p* < 0.0005 for linear trend for both mean values and proportions, except *p* = 0.001 for ALT data on proportions among women

**Fig. 2:**
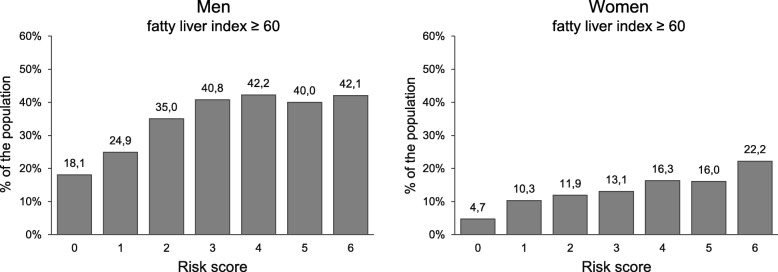
The occurrence of abnormal FLI in groups classified according to alcohol use, smoking and physical activity as risk factors in men and women as follows: Alcohol consumption, 0 = no consumption; 1 = alcohol consumption 1–14 (men) or 1–7 (women) drinks/week; 2 = alcohol consumption exceeding 14 drinks (men) or 7 drinks (women) per week Smoking, 0 = no smoking, 1 = 1–19 cigarettes/day, 2 = ≥ 20 cigarettes/day. BMI, 0 = BMI < 25; 1 = BMI ≥ 25 and < 30; 2 = ≥ 30. Physical activity, 0 = physical activity more than 4 h/week; 1 = physical activity 0.5–4 h/week; 2 = physical activity less than 30 min/week. The sum of the scores yielded a total risk factor number, with higher scores indicating an unhealthier lifestyle (maximum = 6)

Figure 2 demonstrates the rates of abnormal FLI results in the study population classified according to risk factor scores based on alcohol consumption, smoking and physical inactivity as independent individual components of risk factor classification (score range 0–6). In comparisons to those with zero risk factors, a significant increase in the occurrence of abnormal FLI was found in those with one or more risk factors *(p* < 0.0005 for all comparisons). In these analyses, the FLI responses were also found to be more pronounced among men. The data on multinomial logistic regression analysis for increased FLI, as adjusted for BMI, age and coffee consumption, are summarized in Table 3. The risk score status was associated with significant increases in ORs for FLI 60 and above in the groups with one or more risk factors. The most striking influences on the likelihood of abnormal FLI were observed for lack of physical activity *(p* < 0.0005 for both genders) and alcohol consumption exceeding current low risk drinking limits in men (14 drinks per week) *(p* < 0.0005) (Table 4).

**Table 3:** Odds ratios for abnormal FLI according to the individual number of lifestyle risk factor scores, as derived from multinomial logistic regression analysis, adjusted for BMI, age and coffee consumption

	Men		Women	
	FLI ≥ 30 and < 60	FLI ≥ 60.0	FLI ≥ 30 and < 60	FLI ≥ 60.0
**Risk score**	**OR (95% CI)**	**OR (95% CI)**	**OR (95% CI)**	**OR (95% CI)**
0	1.0	1.0	1.0	1.0
1	1.8 (1.3–2.6)	1.7 (1.0–2.8)	0.8 (0.6–1.2)	2.2 (1.1–4.5)
2	2.3 (1.7–3.3)	3.6 (2.2–5.9)	1.2 (0.8–1.7)	3.5 (1.7–7.2)
3	2.9 (2.0–4.1)	6.6 (4.0–11.0)	1.8 (1.2–2.7)	6.4 (3.0–13.5)
4	4.6 (3.0–6.9)	13.5 (7.7–23.7)	2.1 (1.3–3.4)	9.6 (4.1–22.9)
5	7.4 (4.5–12.1)	29.7 (15.5–57.1)	3.5 (1.9–6.6)	26.3 (8.9–77.8)
6	6.2 (3.0–12.7)	28.1 (11.2–70.9)	9.3 (2.3–37.6)	32.8 (11.6–92.3)

*FLI* fatty liver index, *OR* odds ratio

**Table 4:** Individual impacts of lifestyle factors on fatty liver index in multivariate binary logistic regression analysis

	Men		Women	
	Adjusted OR (95% CI)	p^*^	Adjusted OR (95% CI)	p^*^
Physical activity per week		< 0.0005		< 0.0005
> 4 h	1.0		1.0	
0.5–4 h	2.54 (2.21–2.92)		2.53 (1.99–3.22)	
< 30 min	2.78 (2.35–3.28)		3.82 (2.94–4.96)	
Standard drinks per week		< 0.0005		0.086
none	1.0		1.0	
1–14 (men) or 1–7 (women)	1.01 (0.88–1.15)		0.84 (0.72–0.99)	
> 14 (men) or > 7 (women)	1.81 (1.53–2.15)		1.02 (0.80–1.30)	
Cigarettes per day		0.047		0.185
none	1.0		1.0	
1–19	0.84 (0.73–0.98)		0.88 (0.7–1.08)	
≥ 20	0.88 (0.75–1.04)		1.25 (0.88–1.78)	

^*^likelihood ratio test

In the analyses of correlations between FLI and the various study parameters, significant correlations were found to emerge between FLI and serum ALT (*R*_*s*_ = 0.512 for men; *R*_*s*_ = 0.322 for women) and CRP (*R*_*s*_ = 0.429 for men; *R*_*s*_ = 0.479 for women) *(p* < 0.001 for all comparisons).

## Discussion

The present findings indicate that combinations of unfavorable determinants in lifestyle markedly increase the risk for fatty liver, as assessed using a recently developed predictor algorithm, FLI. The rather linear association between abnormal FLI and combined lifestyle risk factor status supports the view that significant benefits on liver health could be gained from simultaneous adherence to multiple low-risk lifestyle-related factors and from systematic behavior change support systems for individuals presenting with high-risk lifestyles [1–4, 7, 8]. Based on recent population surveys successful lifestyle interventions could lead to a striking reduction in mortality from both hepatic and extrahepatic causes [1, 2, 4, 17, 19]. Current data indicates that FLI, a non-invasive biomarker of steatosis, could perhaps be used as a clinical tool for patient guidance and motivation during interventions aimed at maintaining long-term lifestyle changes that promote the loss of liver fat.

Fatty liver is currently a highly common condition in high income countries being estimated to affect at least 25–30% of adults in general population and over 70% of those with gross obesity or diabetes [23–25]. Therefore, greater awareness of this phenomenon is important to prevent a looming public health crisis. Building of excess fat in liver cells has been regarded as the hepatic manifestation of the metabolic syndrome, which associates with cerebro- and cardiovascular disease risks, tissue triglyceride deposition, hyperinsulinemia and insulin resistance [10, 19, 23, 26–28]. Therefore, new non-invasive tools for detecting hepatic steatosis in an early phase are needed to prevent progression of liver disease and associated metabolic comorbidities. Although the FLI algorithm has recently been shown to improve the identification of fatty liver when compared with other non-invasive methods [11, 20, 29–31], as yet, only few studies have been available on the clinical applications of FLI or the effects of lifestyle factors on FLI.

Alcohol drinking, cigarette smoking, and physical inactivity are currently the main modifiable high-risk determinants of lifestyle [1]. The present findings indicate that each of these components and especially their co-existence increase the risk of metabolic aberrations in the liver. In obese individuals or in smokers, regular alcohol drinking even in relatively modest amounts may increase the risk for abnormal liver enzyme activities [6, 15, 18, 32]. The combined triggers from multiple unfavorable lifestyle factors may also stimulate inflammation and lead to progression of fibrosis [6, 12, 15, 16, 33]. The present findings also lend support to the view that no safe limit of alcohol consumption in relation to the risk of progression of non-alcoholic fatty liver disease (NAFLD) can be defined. Thus, questioning such patients about alcohol intake and other factors of lifestyle warrants further attention. Previous findings have indicated that there may be common pathogenic features in lifestyle-related disease manifestations, including systemic inflammatory response, oxidative stress and altered fatty acid metabolism [9, 34–36]. Therefore, use of FLI together with biomarkers reflecting the above mentioned pathophysiological pathways could also help in elucidating the primary mechanisms of fatty deposition in various behavioral phenotypes. Recently, a link between hepatic and extrahepatic manifestations of fatty liver have been proposed based on findings indicating that LDL oxidation in coronary atherosclerotic plaques can be boosted by the action of GGT enzyme, which is also a key mediator of oxidative stress [37, 38]. There may also be an interplay between oxidative stress and inflammation [13, 39–41]. In line with this view, current data shows that abnormalities in serum CRP, a biomarker and important regulator of inflammation also coincide with the burden of high-risk lifestyle factors and abnormalities in FLI.

Lack of physical activity has recently been recognized as an increasingly important lifestyle-associated contributor to poor health [42, 43]. Spending more time in sedentary behaviors associates with a wide variety of adverse health outcomes, including cardiovascular diseases, diabetes and carcinogenesis [1, 44–47]. The present data shows that physical inactivity is also a major independent contributor of abnormal FLI. Those with moderate and vigorous physical activity show markedly lower odds for fatty liver than those with sedentary activity. Sufficient doses of physical exercise could also have a major impact in reducing the adverse metabolic effects of unfavorable lifestyle. Regular physical activity may also be expected to lead to significant long-term health benefits in reducing hepatic steatosis and insulin resistance [35, 45, 48–50]. In accordance with this view, moderate or vigorous physical activity were recently shown to reduce fat, inflammation and oxidative stress in the liver even in cases without any notable changes in BMI status [35].

Previous studies have shown that Western diet characterized by high fat, high carbohydrate and insufficient vitamin intake may provide triggers for insulin resistance and associated hepatotoxicity [14, 46, 51–56]. On the other hand, adherence to a healthy diet has recently been emphasized among the first-line treatment options for NAFLD [52, 57]. Unfortunately, in this work information on the exact compositions of the diet were not available. A large body of evidence has supported the view that nutrients rich in antioxidants show an inverse association with the risk of mortality due to NAFLD [52]. Interestingly, consumption of coffee, which is a rich source of antioxidants, has been previously associated with a reduced risk for liver cirrhosis and liver enzyme elevations in alcohol consumers [58, 59]. Coffee intake has also been suggested to be inversely related with the risk of NAFLD possibly by modulating pathways of the gut-liver axis [60]. In the present population, the lifestyle risk factor score was found to correlate positively with coffee intake, which was explained by a high prevalence of coffee drinking among smokers [61]. The question whether and how coffee consumption could exert protective effects towards the oxidative stress induced by combined lifestyle associated risk factors remains, however, unknown.

A major strength of this study is the large sample size of over 12,000 participants with a comprehensive assessment of the relationships between FLI, other laboratory markers and lifestyle-related risk factors. Although the present material was collected from different geographical areas in Finland, the population represents a Caucasian population with a high degree of environmental and genetic homogeneity. Based on previous evidence indicating profound gender-related differences in susceptibilities for liver disease, we have also included separate analyses for men and women. In accordance with recent findings from an animal model for NAFLD [62], our data suggests that alterations in liver enzymes and lipid status among men may occur relatively early in the sequence of events leading abnormal FLI. However, the changes in CRP, a biomarker of inflammation, in response to combined life style risk factors appeared to be more pronounced among women.

The main limitation of the study is the cross-sectional setting and lack of follow-up data to address possible causal relationships. The data on lifestyle determinants were based on self-reports and therefore we cannot rule out the possibility of recall bias or underreporting especially concerning the data reflecting socially less desirable behaviors, such as alcohol intake. Lack of detailed information on the patterns of diet may also be kept as a limitation of the study. Therefore, future longitudinal studies are needed to examine causal relationships between combinations of life style risk factors and fatty change in the liver. The possible role of FLI as a clinical tool for supporting behavior changes in NAFLD patients also warrant future studies in large materials. It should further be emphasized that although elevated blood glucose levels is known to be an important determinant of metabolic health in both normal weight and obese subjects [63], in this study data on simultaneous measurements of fasting blood glucose levels were not available. The occurrence of abnormal blood glucose status is, however, unlikely to create a significant confounding factor in the present analyses since we excluded all subjects who had been previously diagnosed with diabetes or had shown abnormal results in oral glucose tolerance tests.

## Conclusions

Taken together, current data demonstrates distinct relationships of lifestyle-related risk factors and fatty liver, which should be implicated in recommendations aimed at promoting liver health. The data also emphasizes the possibility of using FLI algorithm as a non-invasive clinical tool for providing feedback in approaches to reduce the number of unfavorable lifestyle risk factors and to prevent morbidity and mortality resulting from fatty liver disease and associated metabolic comorbidities. Interestingly, recent studies have indicated that FLI could also serve as a risk predictor for extrahepatic complications, such as chronic kidney disease [64].

## Data Availability

THL Biobank administrates and grants access to the FINRISK data to research projects that are of high scientific quality and impact, are ethically conducted, and that correspond with the research areas of THL Biobank. All data are available for application at https://thl.fi/en/web/thl-biobank/for-researchers/sample-collections/the-national-finrisk-study-1992-2012. The name of dataset is the National FINRISK Study 1992–2012.

## Abbreviations

ALT: Alanine aminotransferase
BMI: Body mass index
CRP: C-reactive protein
FLI: Fatty liver index
GGT: Gamma-glutamyl transferase
HDL: High-density lipoproteins
LDL: Low-density lipoprotein
NAFLD: Non-alcoholic fatty liver disease
NASH: Non-alcoholic steatohepatitis
